# Forskolin Protected against Streptozotocin-Induced Diabetic Cardiomyopathy via Inhibition of Oxidative Stress and Cardiac Fibrosis in Mice

**DOI:** 10.1155/2021/8881843

**Published:** 2021-01-29

**Authors:** Xu Zhang, Pei-Xiong Ke, Xun Yuan, Gui-Ping Zhang, Wen-Liang Chen, Gen-Shui Zhang

**Affiliations:** ^1^Key Laboratory of Molecular Clinical Pharmacology & Guangzhou Institute of Cardiovascular Disease, Guangzhou Medical University, Guangzhou, Guangdong 511436, China; ^2^Department of Biology, York University, 4700 Keele Street, Toronto, ON, Canada M3J 1P3

## Abstract

**Background:**

Diabetic cardiomyopathy is one of the cardiac complications in diabetes patients, eventually resulting in heart failure and increasing morbidity and mortality. Oxidative stress is a critical pathological feature in diabetic hearts, contributing to the development of DCM. Forskolin (FSK) was shown to reduce oxidative stress. This study was aimed at investigating the effects of FSK on diabetic hearts and the relevant molecular mechanisms.

**Methods:**

Streptozotocin- (STZ-) induced diabetes in mice was treated with FSK through intraperitoneal injection. Cardiac functions were evaluated by echocardiography. Hematoxylin-eosin and Masson trichrome staining was employed to determine heart morphological changes and cardiac fibrosis, respectively. Cardiac fibrosis-related markers were detected by western blot. Superoxide dismutase activity, reduced/oxidized glutathione ratio, and malondialdehyde concentration in left ventricles were determined using respective commercial kits.

**Results:**

Abnormal cardiac diastolic dysfunction and cardiac fibrosis were observed in diabetic hearts. FSK treatment significantly improved the cardiac diastolic function and attenuated the abnormal morphological change in diabetic hearts. Moreover, FSK treatment in diabetic mice decreased the expression of fibronectin, collagen I, TGF-*β*, and *α*-SMA and reduced myocardial fibrosis. Furthermore, we observed that FSK significantly blocked oxidative stress in diabetic hearts.

**Conclusions:**

Our study demonstrates that FSK protects against the development of DCM in STZ-induced diabetes in mice. Our study suggests that FSK might be a potential target for drug development in treating DCM.

## 1. Introduction

The global incidence of diabetes mellitus (DM) is predicted to increase to 380 million and approximately 700 million by 2025 and 2045, respectively [1, 2]. The prevalence of diabetic cardiomyopathy (DCM) in patients of type 1 diabetes mellitus (T1DM) and type 2 diabetes mellitus (T2DM) was 14.5% and 35.0%, respectively [3, 4]. Diabetic patients exhibited a higher risk of developing heart failure resulting from DCM than sex-matched controls [5]. It remains urgent to develop effective drugs to prevent and treat this heart complication in diabetes patients.

DCM is characterized by abnormal myocardial structure and cardiac dysfunction, without observing other cardiac risk factors, such as hypertension and macrovascular coronary diseases [6]. Myocardial fibrosis is one of the critical pathological changes in diabetic hearts. Most diabetes patients do not exhibit a significant reduction in cardiac systolic function in the early stage but show diastolic dysfunction which is the earliest clue of heart failure [7]. Our previous study consistently showed abnormal cardiac diastolic function and myocardial fibrosis in streptozotocin-induced diabetes in mice [8]. Hypoglycemic drugs are widely administrated to control blood glucose levels in diabetes patients. However, intensified glycemic control did not show significant outcomes in cardiovascular complications of diabetes [9, 10], suggesting that blood glucose-independent factors contribute to DCM. Currently, the therapeutic strategy for the treatment of DCM includes improving insulin resistance, lowering inflammation, and reducing oxidative stress [11].

Cardiac oxidative stress is an initial feature of DCM, characterized by a serious imbalance between reactive oxygen species (ROS) and antioxidants [12]. Malondialdehyde (MDA) concentration increased in diabetes resulting in excessive ROS generation and oxidative damage in organs [13]. In contrast, high levels of superoxide dismutase (SOD) and reduction of glutathione (GSH) scavenged ROS and thus improved diabetic heart function [14]. Blocking ROS generation or enhancing antioxidant ability reduced oxidative stress in diabetes, thereby inhibiting DCM development [15, 16]. Thus, restraining oxidative stress serves as a therapeutic strategy for the prevention and treatment of DCM.

**Figure 1:**
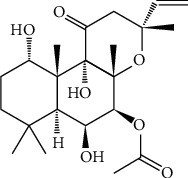
The chemical structure of forskolin (downloaded from https://www.wikidata.org/wiki/Q412747).

Forskolin (FSK) (Figure 1) is a natural product derived from the root of *Coleus forskohlii* with a long history in traditional Ayurvedic medicine [17]. As an adenylate cyclase activator, FSK is widely used to increase intracellular cAMP levels. Meanwhile, FSK also showed an intense antioxidant ability, which significantly inhibited lipid peroxides in human red blood cells induced by high glucose [18]. Besides, FSK attenuated the renal damage in diabetic rats induced by low-dose streptozotocin (STZ) injection and high-fat diets [19].

This study hypothesized that FSK could improve diabetic heart function through its antioxidative stress ability. We treated STZ-induced diabetic mice with FSK through intraperitoneal injection and evaluated the effects of FSK on cardiac function, structural changes, cardiac fibrosis, and oxidative stress.

## 2. Materials and Methods

### 2.1. Drugs

Streptozotocin (purity ≥ 98%) was purchased from Sigma-Aldrich Co. (USA). Forskolin (purity ≥ 99.0%) was purchased from Target Molecule Corp. (USA). Forskolin stock solution (20 mg/ml) was prepared with pure dimethyl sulfoxide (DMSO, Sigma-Aldrich Co., USA) and stored at -20°C.

### 2.2. Experimental Animals and Procedures

Experimental protocols were approved by the Animal Care and Use Committee of Guangzhou Medical University, China (no. 2010-225). Male C57BL/6 wild-type mice (6-8 weeks old) were purchased from the Guangdong Experimental Animal Center, China. All mice were maintained at standard laboratory conditions (temperature at 20-24°C, humidity at 60%, and 12-hour light/dark cycle). Mice freely accessed food and water. Diabetes mice were induced by intraperitoneal injection of streptozotocin (STZ, 60 mg/kg/day) for six consecutive days while the control mice received equivalent volumes of citrate buffer. Mice with fasting blood glucose concentrations > 16.7 mmol/l seven days after STZ injection were considered having diabetes. Eight weeks after the establishment of diabetes, mice were randomly assigned to three groups (*n* = 8 in each group): (1) control group (Con), (2) DM group in which diabetic mice were treated with vehicle, and (3) FSK group in which diabetic mice were treated with FSK (DM+FSK, 2 mg/kg/d, i.p.) for four weeks. The dose of FSK used in this study was based on a previously published study [20]. All diabetic mice survived for the whole experimental duration.

### 2.3. Doppler Echocardiography

Mouse cardiac function was determined at the study endpoint by using the Vevo 2100 echocardiography system with an 18-38 MHz linear transducer (Visual Sonics, Toronto, Canada). Mice were anesthetized by using 1.0%-1.5% isoflurane mixed with 100% oxygen. Left ventricular systolic function was measured by M-mode ultrasound through the parasternal short-axis view at the papillary muscle level. Left ventricular ejection fraction (EF) and left ventricular fractional shortening (FS) were measured. Diastolic function parameters were measured using pulse-wave Doppler, including maximum flow velocity of peak *E* wave (*E*-wave velocity), maximum flow velocity of peak *A* wave (*A*-wave velocity), mitral valve descending time (MV decel time), and isovolumetric relaxation time (IVRT) and *E*/*A* ratio. All values were obtained from five continuous cardiac cycles.

### 2.4. Tissue Preparation

The body weight of each mouse was obtained after accomplishing FSK treatment. All mice were then anesthetized with isoflurane and sacrificed through cervical dislocation. Hearts were rapidly excised and placed into phosphate buffer saline (pH 7.4). The left ventricles were rapidly frozen in liquid nitrogen and stored at -80°C until use.

### 2.5. Cardiac Oxidative Stress Assays

Hearts were excised and washed with cold PBS and placed into ice-cold RIPA lysis buffer containing phenylmethanesulfonyl fluoride (PMSF) (Beyotime Institute of Biotechnology, Shanghai, China) to grind homogenate into 10% homogenate. The homogenate was centrifuged once at 14,000 rpm at 4°C for 30 min and debris discarded. The protein concentration of the supernatant was determined by using the BCA Protein Assay Kit (Thermo Fisher Scientific, USA). The supernatant lysate was used for the detection of superoxide dismutase (SOD), reduced/oxidized glutathione (GSH/GSSG), and malondialdehyde (MDA) content.

The SOD activity in left ventricle lysate homogenates was determined by using xanthine oxidase assay kits (A001, Nanjing Jiancheng Bioengineering Institute, China). The samples were mixed with ddH_2_O, enzyme solution, and diluting buffer according to the product's instructions, and the mixed solution was incubated at 37°C for 20 min. Then, the optical density of solution was measured at 450 nm using an automatic microplate reader. SOD activity was expressed as units per milligram protein.

The contents of total glutathione (GSH) and oxidized glutathione (GSSG) in myocardial tissue were determined using a total glutathione/oxidized glutathione assay kit (A061, Nanjing Jiancheng Bioengineering Institute, China). According to the kit instructions, 10 *μ*l or 100 *μ*l sample was mixed with respective substrate solutions for GSH and GSSG measurement, respectively. After incubation, the solution OD value was read by using an automatic microplate reader at 405 nm. The GSH/GSSG ratio was presented.

MDA concentration was determined by using a thiobarbituric acid assay kit (A003, Nanjing Jiancheng Bioengineering Institute, China). This MDA assay kit utilizes the thiobarbituric acid (TBA) method. According to the instructions, the samples were mixed with TBA solution and incubated at 95°C for 40 min. Then, the mixture was cooled down under running water followed by centrifugation at 3500 rpm for 10 min. Finally, the optical density of the reaction solution was measured using an automatic microplate reader at 532 nm. The level of MDA was expressed as nanomoles per milligram protein. All procedures followed the manufacturer's instructions.

### 2.6. Hematoxylin-Eosin (H-E) Staining and Masson Trichrome Staining

Mouse hearts were fixed with 4% paraformaldehyde solution for 24 hours and used for paraffin-embedded tissue slice (5 *μ*m). The slices were baked at 60°C for 1 hour, routinely dewaxed, rinsed, and stained with H-E and Masson trichrome staining, respectively. Samples were imaged under a microscope with 400x magnification.

### 2.7. Western Blot

Left ventricular tissues were lysed in RIPA buffer containing phenylmethanesulfonyl fluoride (PMSF). Protein concentration was measured by using the BCA Protein Assay Kit (Thermo Fisher Scientific, USA). Protein samples (30 *μ*g) were separated by 8% or 10% SDS-PAGE, followed by transferring to the PVDF membrane (Millipore, Bedford, MA, USA). After blocking with 5% skimmed milk diluted in 1x Tris-buffered saline containing 0.1% Tween-20 (TBST) for 1 h, the blotted membrane was incubated with corresponding primary antibodies (diluted with 5% BSA in TBST) at 4°C overnight, including anti-fibronectin (FN, Boster Biological Technology, Wuhan, China), anti-transforming growth factor-*β* (TGF-*β*, Abcam, USA), anti-collagen I (Bioworld Technology, USA), anti-*β*-tubulin (Cell Signaling Technology, USA), and anti-*α*-smooth muscle actin (*α*-SMA, Bioworld Technology, USA). Then, corresponding secondary anti-rabbit HRP-linked antibodies (Cell Signaling Technology, USA) were incubated with the membrane at room temperature for one hour. Blotting bands were visualized using an enhanced chemiluminescence reagent kit (Cell Signaling Technology, USA), and the gray values were analyzed using the Quantity One software (Bio-Rad, USA).

### 2.8. Statistical Analysis

Data were expressed as means ± SD. One-way ANOVA with the Student-Newman-Keuls (SNK) test as a post hoc test was used to compare the difference in more than two groups. Statistical analyses were performed using SPSS software package 22.0 for Windows. A *P* value of less than 0.05 was considered statistically significant.

## 3. Results

### 3.1. FSK Improved the Left Ventricle Diastolic Function in Diabetic Mice

**Figure 2:**
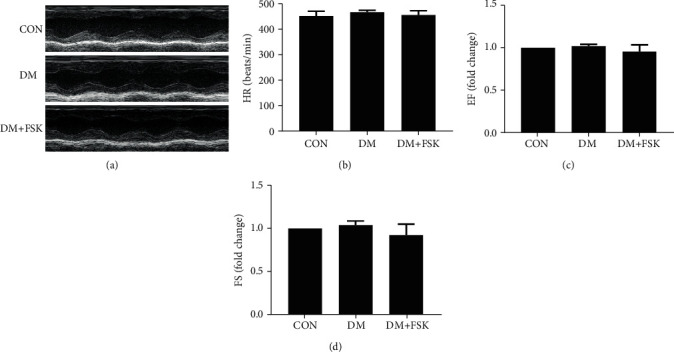
Effects of FSK treatment on the systolic function in diabetic mice. Ventricular function was determined using echocardiography. The systolic function in STZ-induced diabetic mice was comparable between groups. (a) Representative M-mode echocardiographic images of hearts. (b) Heart rate (HR). (c) Ejection fraction (EF). (d) Fraction shortening (FS). Data presented as means ± SD (*n* = 6).

**Figure 3:**
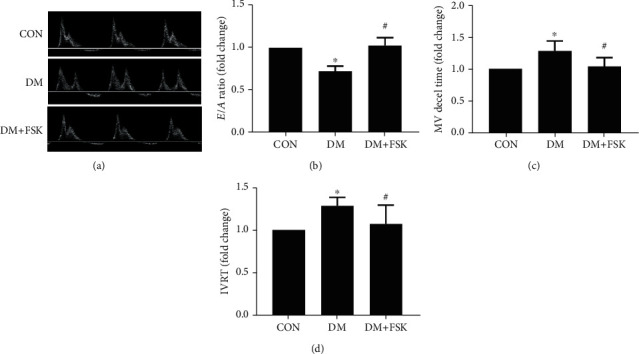
Effects of FSK treatment on the diastolic function in diabetic mice. In the DM group, the *E*/*A* ratio was significantly decreased, while mitral valve descending time and isovolumetric relaxation time were significantly increased. These indexes were significantly ameliorated by FSK treatment. (a) Representative images of *E* and *A* waves. (b) Analysis results of the *E*/*A* ratio. (c) Analysis results of mitral valve (MV) descending time. (d) Analysis results of isovolumetric relaxation time (IVRT). Data presented as means ± SD (*n* = 6). ^∗^*P* < 0.05 vs. control group and ^#^*P* < 0.05 vs. DM group.

Firstly, we measured cardiac function in diabetic mice by echocardiography. As shown in Figure 2, diabetic mice did not show a significant change in left ventricular systolic function at 12 weeks compared with the control mice (*P* > 0.05). FSK treatment did not significantly affect the systolic function in diabetic mice. However, we observed that diastolic function significantly decreased in the DM group showing a lower *E*/*A* ratio (0.72 ± 0.06-fold of control) but higher levels of mitral valve descending time (MV decel time 1.28 ± 0.16-fold of control) and IVRT (1.29 ± 0.10-fold of control) than that in the control group, which were significantly reversed by FSK treatment (1.03 ± 0.09-fold of control, 1.04 ± 0.14-fold of control, and 1.07 ± 0.22-fold of control, respectively; *P* < 0.05) (Figure 3).

### 3.2. FSK Treatment Inhibited Abnormal Morphological Alteration and Cardiac Fibrosis in the Hearts of DM Mice

**Figure 4:**
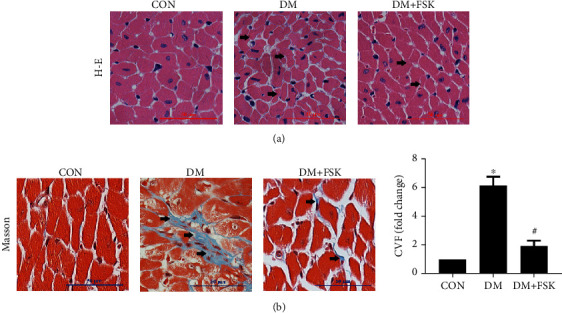
Effects of FSK treatment on diabetic cardiac structural changes. FSK treatment significantly ameliorated myocardial structural damage and collagen deposition. (a) Representative images of H-E-stained left ventricle (LV) sections. (b) Representative images of Masson's trichrome-stained LV sections and the analysis result of collagen volume fraction (CVF). All images were obtained in the microscope with original 400x magnification (scale bar = 50 *μ*m). Data presented as means ± SD (*n* = 6). ^∗^*P* < 0.05 vs. control group and ^#^*P* < 0.05 vs. DM group.

We then determined the effects of FSK on the heart morphological changes and cardiac fibrosis using H-E staining and Masson trichrome staining, respectively. As shown in Figure 4, H-E staining showed that the myocardium displayed remarkable structural abnormalities in the DM group: broken myocardial fibers, deranged myocyte structures, larger cell gap, and the existence of necrotic myocytes; in contrast, FSK treatment significantly alleviated these pathological changes (indicated by arrows) in the hearts of DM mice (Figure 4(a)). Masson trichrome staining showed that collagen deposition predominantly in the interstitial area was increased in diabetic mice (6.25 ± 0.68-fold of control, *P* < 0.05), which was significantly ameliorated by FSK treatment (1.95 ± 0.45-fold of control, *P* < 0.05) (Figure 4(b)). These results suggest that FSK treatment significantly alleviates cardiac fibrosis in DM mice.

### 3.3. FSK Treatment Attenuated FN, Collagen I, TGF-*β*, and *α*-SMA Expressions in DM Mouse Hearts

**Figure 5:**
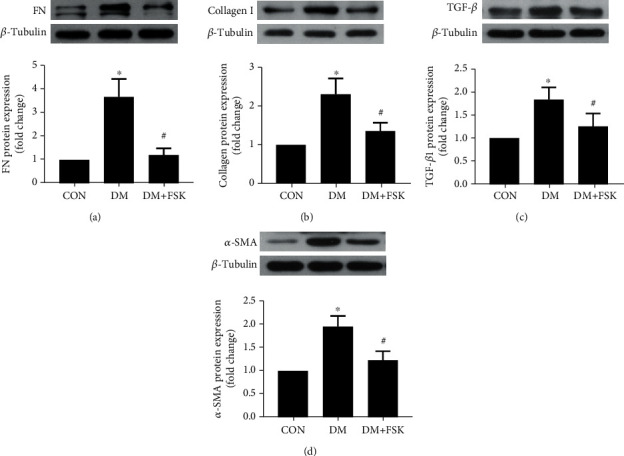
Effects of FSK treatment on the expression of cardiac fibrosis-related makers in diabetic hearts. The protein expression of FN, collagen I, TGF-*β*, and *α*-SMA was significantly decreased by FSK treatment in a diabetic heart. Representative images of western blot and the corresponding analysis results of FN (a), collagen I (b), TGF-*β* (c), and *α*-SMA (d) were shown. Data presented as means ± SD (*n* = 5). ^∗^*P* < 0.05 vs. control group and ^#^*P* < 0.05 vs. DM group.

Next, we further used western blot to determine the effect of FSK on cardiac fibrosis-related protein expression. As shown in Figure 5, the expression of fibrosis markers, including FN (Figure 5(a)), collagen I (Figure 5(b)), TGF-*β* (Figure 5(c)), and *α*-SMA (Figure 5(d)), was significantly higher in the DM group (3.69 ± 0.76-fold of control, 2.31 ± 0.41-fold of control, 1.82 ± 0.26-fold of control, and 1.96 ± 0.23-fold of control, respectively) than in the control group (*P* < 0.05). FSK treatment significantly reduced the protein expression of these markers in diabetic hearts (1.21 ± 0.29-fold of control, 1.36 ± 0.21-fold of control, 1.26 ± 0.28-fold of control, and 1.24 ± 0.19-fold of control, respectively; *P* < 0.05).

### 3.4. FSK Treatment Did Not Affect Blood Glucose Level and Body Weight in DM Mice

**Figure 6:**
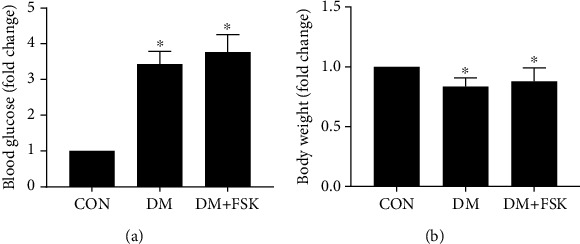
Effects of FSK treatment on blood glucose and body weight in diabetic mice. FSK treatment did not significantly affect both blood glucose (a) and body weight (b) in diabetic mice. Data presented as means ± SD (*n* = 7). ^∗^*P* < 0.05 vs. control group.

We then explored whether FSK treatment protected against diabetic cardiomyopathy by lowering blood glucose. As shown in Figure 6(a), fasting blood glucose significantly increased in diabetic mice compared with control mice (27.67 ± 1.18 mmol/L in diabetic mice vs. 8.06 ± 0.40 mmol/L in control mice; *P* < 0.05), whereas the blood glucose level in the DM+FSK group (30.38 ± 1.64 mmol/L) was similar to that in the DM group (*P* > 0.05). The body weight in the DM group was significantly reduced compared with that in the control group (20.86 ± 0.71 g in diabetic mice vs. 25.01 ± 0.86 g in control mice; *P* < 0.05), while the body weight between the DM group and DM+FSK group (22.10 ± 1.07 g) was comparable (*P* > 0.05) (Figure 6(b)).

### 3.5. FSK Treatment Reduced Oxidative Stress in Diabetic Hearts

**Figure 7:**
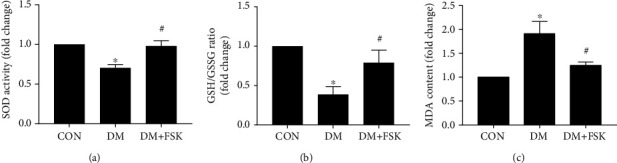
Effects of FSK treatment on cardiac oxidative stress in diabetic mice. FSK treatment significantly ameliorated cardiac oxidative stress in diabetic mice. (a) The analysis results of SOD activity. (b) The analysis results of the GSH/GSSG ratio. (c) The analysis results of MDA contents. Data presented as means ± SD (*n* = 5). ^∗^*P* < 0.05 vs. control group and ^#^*P* < 0.05 vs. DM group.

As shown in Figures 7(a) and 7(b), SOD activity and the GSH/GSSG ratio, indicators of antioxidants, significantly decreased in the DM group (0.70 ± 0.04-fold of control and 0.39 ± 0.10-fold of control, respectively) while FSK treatment significantly inhibited these changes (0.98 ± 0.07-fold of control and 0.79 ± 0.16-fold of control, respectively; *P* < 0.05). In contrast, MDA content was significantly increased in DM mice (1.92 ± 0.26-fold of control; *P* < 0.05), whereas it was significantly blocked by FSK treatment (1.25 ± 0.07-fold of control; *P*<0.05) (Figure 7(c)). These results suggest that FSK treatment inhibits oxidative stress in diabetic hearts.

## 4. Discussion

Our present study highlights the critical role of forskolin (FSK) in improving cardiac diastolic function and suppressing cardiac fibrosis in STZ-induced diabetic mice. Our findings showed that FSK significantly reduced oxidative stress, thereby contributing to its cardiac protective effects in diabetic mice.

Diabetic patients exhibit a higher risk of heart failure than nondiabetic individuals, leading to increased morbidity and mortality [11]. DCM was defined in 2013 as a clinical manifestation characterized by ventricular dysfunction with the absence of hypertension and coronary atherosclerosis in diabetes patients [21, 22]. The early stage of diabetic cardiomyopathy is a subclinical period, showing normal ventricular systolic function but impaired diastolic dysfunction associated with cardiac fibrosis. Diabetes patients with DCM eventually advance to heart failure with limited ejection fraction. Our study is consistent with previous studies [23, 24]. We found that STZ-induced diabetic mice displayed unchanged ejection fraction and fraction shortening at 12 weeks after diabetes establishment. The diabetic mice developed an abnormal ventricular diastolic function showing a decreased *E*/*A* ratio but increased mitral valve descending time and isovolumetric relaxation time. Besides, dramatic cardiac fibrosis was observed in diabetic hearts, exhibiting upregulation of FN, collagen I, TGF-*β*, and *α*-SMA. These results demonstrate that diabetic mice induced by STZ could serve as a convincible animal model for diabetic cardiomyopathy research.

FSK was shown to lower fasting glucose in STZ-induced diabetes in rats [25]. It remained unknown if FSK regulates diabetic heart function. Our study showed that FSK treatment significantly attenuated abnormal cardiac structural change and improved ventricular diastolic function in STZ-induced diabetic mice. Moreover, we also observed that FSK treatment reduced cardiac fibrosis in diabetic mice by reducing the expression of FN, collagen I, TGF-*β*, and *α*-SMA, suggesting that FSK treatment significantly protects against diabetic cardiomyopathy in the early stage. However, during the experimental period, we did not observe that fasting glucose and body weight were affected by FSK treatment in STZ-induced diabetes. In contrast, oral administration of FSK with a dose of 6 mg/kg for eight weeks was found to reduce fasting glucose in both normal control mice and STZ-induced diabetic mice [19, 25]. Compared with the above studies, we administered FSK with a shorter time and a lower dose, explaining the discrepancy of blood glucose results. Our data indicate that FSK's protective effects on the diabetic heart are not dependent on blood glucose. We believe that chronic administration of FSK resulting in a hypoglycemic effect would provide an extra beneficial outcome for diabetic heart treatment, worthy of future investigation.

Animal and clinical studies have pointed out that oxidative stress played a critical role in the development of diabetic cardiomyopathy [26–28]. The decrease in endogenous antioxidant capacity in the myocardium leads to oxidative stress accounting for diabetic cardiomyopathy [29]. Suppression of oxidative stress induced by hyperglycemia is beneficial for the treatment of diabetic cardiomyopathy [15]. FSK was shown as a powerful antioxidant, and FSK oral administration with either dose of 20 mg/kg or 30 mg/kg significantly reduced the level of thiobarbituric acid reactive substances (TBARS), an oxidative stress marker, in kidneys in diabetic rats induced by STZ and feeding of high-fat diets [19]. Consistent with other studies, we also found that FSK treatment significantly attenuated oxidative stress in diabetic hearts.

Our study did not exclude other potential mechanisms accounting for FSK's protective effects against diabetic cardiomyopathy in STZ-induced diabetic mice. For example, FSK exhibited anti-inflammatory effects [30], one of the major harmful factors for diabetic cardiomyopathy. Another limitation of this study is that we did not verify the observed effects of FSK in type 2 diabetes animal models, which requires future investigation.

The present study collectively provides experimental evidence that FSK protects against diabetic cardiomyopathy in STZ-induced diabetic mice with an underlying molecular mechanism involved in suppressing oxidative stress. Our study demonstrates that FSK might serve as a potential target for drug development to treat diabetic heart complications.

## Data Availability

Data is available on request through the corresponding author Dr. Zhang (zhanggs99@hotmail.com).
